# Do investments in sport pay off: a systematic review and exploratory quasi meta-analysis of social return on investment in sport and physical activity

**DOI:** 10.1186/s12966-026-01957-2

**Published:** 2026-07-27

**Authors:** Ágoston Koch, Erika Kendelényi-Gulyás, Tamás Sterbenz, Gergely Csurilla

**Affiliations:** 1 Sports Economics and Decision Making Research Centre, Hungarian University of Sports Science, Budapest, Hungary; 2 Institute of Economics, ELTE Centre for Economic and Regional Studies, Budapest, Hungary

**Keywords:** Social return on investment, Physical activity, Public health, Systematic review, Exploratory quasi meta-analysis, Social impact assessment

## Abstract

**Background:**

Physical inactivity remains one of the most pressing global health concerns, contributing to millions of deaths each year and placing a considerable financial burden on public health systems through increased healthcare expenditure. Sport and physical activity have been recognised as effective tools for promoting health, strengthening social inclusion, and improving overall well-being. Traditional evaluation approaches, such as cost-benefit and cost-effectiveness analyses, have helped demonstrate economic value but often fail to capture wider social outcomes. The Social Return on Investment (SROI) framework provides a more comprehensive approach by assessing the combined health, economic, and social value generated per unit of investment.

**Methods:**

This systematic review with an exploratory quasi-meta-analytic synthesis examined studies applying the SROI framework to sport and physical activity interventions published between 2018 and 2024. Following PRISMA guidelines, five academic databases and the Social Value UK repository were searched. Studies were included if they reported an SROI ratio for an empirical sport or physical activity intervention. Data were extracted on study and intervention characteristics, outcome domains, and methodological quality, the latter assessed using Krlev et al.’s 12-point framework. Because most studies did not report standard deviations or confidence intervals, formal variance-weighted pooling was not possible. Therefore, descriptive unweighted and sample-size-weighted averages were calculated, supported by sensitivity analyses and exploratory correlation analyses between SROI and study characteristics.

**Results:**

Sixteen studies met the inclusion criteria, providing 17 SROI estimates across interventions ranging from preventive health programmes to sport-for-development initiatives. The median SROI was 5.07:1, with estimates ranging from 0.03:1 to 101.05:1. The unweighted mean was 12.22:1, but this value was strongly influenced by the highest estimate; excluding the 101.05:1 outlier reduced the unweighted mean to 6.67:1 and the sample-size-weighted mean from 7.07:1 to 2.72:1. These findings indicate that median and sensitivity-adjusted estimates provide a more conservative summary of the available evidence.

**Conclusions:**

Overall, the evidence indicates that investment in sport and physical activity generally generates positive social value, often returning several times the initial cost. However, pooled averages are sensitive to extreme SROI values, and large variations across studies highlight the need for greater methodological consistency, clearer reporting, and broader geographical coverage.

**Supplementary Information:**

The online version contains supplementary material available at https://doi.org/10.1186/s12966-026-01957-2.

## Background

Physical inactivity is a significant global public health issue, identified by the World Health Organization (WHO) as the fourth leading cause of death worldwide, accounting for approximately 3.2 million fatalities annually [1, 2]. A sedentary lifestyle greatly increases the likelihood of developing chronic illnesses, such as cardiovascular disease, type 2 diabetes, obesity, osteoporosis, and certain types of cancer [3, 4]. The economic impact is considerable: inactivity leads to increased healthcare costs, places a greater burden on public health systems, and lowers workforce productivity [5].

In response, sports and physical activities have become effective means of promoting public health, enhancing social inclusion, and boosting overall well-being. Regular physical activity improves physical and mental health while also supporting broader social outcomes such as inclusion, community cohesion, and educational attainment [6–14]. As awareness of these benefits grows, policymakers are increasingly pressured to justify investments in sports and physical activity programs through rigorous, evidence-based economic evaluations. Traditional evaluation methods, like cost-effectiveness analysis (CEA) and cost-benefit analysis (CBA), have been crucial in measuring the health and economic returns of interventions [15, 16]. However, in applied sport and physical activity evaluations, these methods often focus on readily quantifiable health and economic outcomes, while the broader societal value—such as social cohesion, cultural identity, and mental well-being— may be less systematically captured.

Social Return on Investment (SROI) has emerged as a novel framework that effectively addresses these shortcomings [17, 18]. Based on social accounting principles, SROI aims to quantify a wide array of outcomes—both concrete and abstract—by creating a ratio of total benefits to total investment [18, 19]. Compared to CEA and CBA, SROI places a more explicit and systematic emphasis on identifying and valuing non-market and social outcomes, thereby offering a more comprehensive perspective on an intervention’s value.

In sport and physical activity settings, SROI has been applied to community programmes, health interventions, and sport-for-development initiatives, often reporting positive returns beyond direct financial gains [20, 21]. Nevertheless, methodological heterogeneity remains substantial, particularly regarding valuation approaches, attribution assumptions, and the treatment of non-market outcomes [19]. Recent efforts to harmonise the assessment of the socio-economic value of sport at the EU level, together with emerging work towards standardising SROI frameworks in physical activity and sport, further highlight the need for consistent and comparable evaluation approaches [22, 23].

In this study, we investigate whether investments in sport and physical activity generally generate positive social returns, and examine which intervention types appear to generate higher social value within the currently available SROI evidence base. More broadly, our aim is to demonstrate how the Social Return on Investment (SROI) framework can capture the multidimensional benefits of physical activity programs and provide policymakers with robust evidence to guide future investment decisions.

We contribute to the existing literature in three main ways. First, we go beyond earlier reviews that relied predominantly on grey literature by systematically including peer-reviewed research published after 2018. Gosselin et al. [19] produced a seminal contribution as the first to conduct a systematic review of SROI studies. At the time, however, suitable peer-reviewed research was unavailable, so they necessarily drew on grey literature. Since then, the volume of peer-reviewed work has grown rapidly, making a fresh reassessment both timely and warranted.

Second, rather than merely replicating previous findings, we explicitly seek to highlight how the evidence base has changed once peer-reviewed studies are incorporated alongside grey literature. Including peer-reviewed literature is essential, as publication type may influence reported findings. While previous evidence-synthesis research has often found larger effects in peer-reviewed studies compared with grey literature [24, 25], SROI evaluations may follow a different pattern because methodological assumptions, valuation approaches, and reporting practices can vary substantially between academic and commissioned grey-literature studies.

Third, using an exploratory quasi meta-analytic approach, we descriptively compare outcomes reported in peer-reviewed and grey-literature studies and test for systematic differences in estimated social returns on investment. Because most studies failed to report SDs/CIs – despite author enquiries – a formal variance-weighted meta-analysis was infeasible. Instead, we report unweighted and sample-size-weighted mean SROI ratios, sensitivity analyses excluding the highest SROI values, and Pearson correlations between SROI and study characteristics with pairwise sample sizes, p-values, and 95% confidence intervals.

This review provides an updated synthesis of the SROI evidence base in sport and physical activity while also highlighting methodological challenges affecting comparability across studies.

## Methods

The systematic review and meta-analysis was conducted according to the Preferred Reporting Items for Systematic Reviews and Meta-analysis (PRISMA) to ensure the quality of the method [26]. The review protocol was registered in PROSPERO (CRD420251147999).

The SROI ratio is constructed by dividing the total monetized social value by the total investment. To ensure a conservative estimate, impact and outcome valuations were adjusted for deadweight, attribution, and displacement. A detailed breakdown of the SROI calculation mechanics and the role of financial proxies is provided in Additional File 5.

### Eligibility criteria

Studies were found eligible when they met the following criteria: [1] study uses SROI framework as the main evaluation method and SROI ratio is reported; [2] sport and/or physical activity is the main intervention component; [3] study is empirical. Inclusion criteria are also presented in Table 1 using PICO framework [27]. Only studies written in English were included in the analysis.

**Table 1:** Inclusion criteria in study selection based on PICO framework

Parameters	Description of detail
Population (P):	Any individuals or groups targeted by the intervention or initiative
Intervention (I):	Physical activity and/or sport programs, interventions, initiatives
Comparison (C):	No intervention or alternative intervention (not PAS)
Outcome (O):	Social return on investment ratio and associated social outcomes
Study design:	SROI framework

### Search strategy

The literature search was conducted in two phases. First, articles in five peer-reviewed databases (SportDiscus, Web of Science, Scopus, PubMed, ScienceDirect) were identified using the same search terms as in Gosselin et al. [19]: (‘social return on investment’ OR ‘SROI’) AND (‘sport’ OR ‘physical activity’). After that, grey literature articles were searched in Social Value UK database. For both peer-reviewed and grey literature articles, search was conducted between January 15th, 2025, and February 15th, 2025. The search was limited to studies published between 2018 and 2024, as the previous systematic review by Gosselin et al. covered studies up to 2018 [19].

Articles identified from different databases were analysed using Rayyan software. Two of the authors independently screened the titles and abstracts of peer-reviewed papers, then final decision was made by the third author in case of conflict. Subsequently, the full texts of the remaining studies were collected and checked for eligibility in accordance with the previously established criteria.

### Study selection

**Fig. 1:**
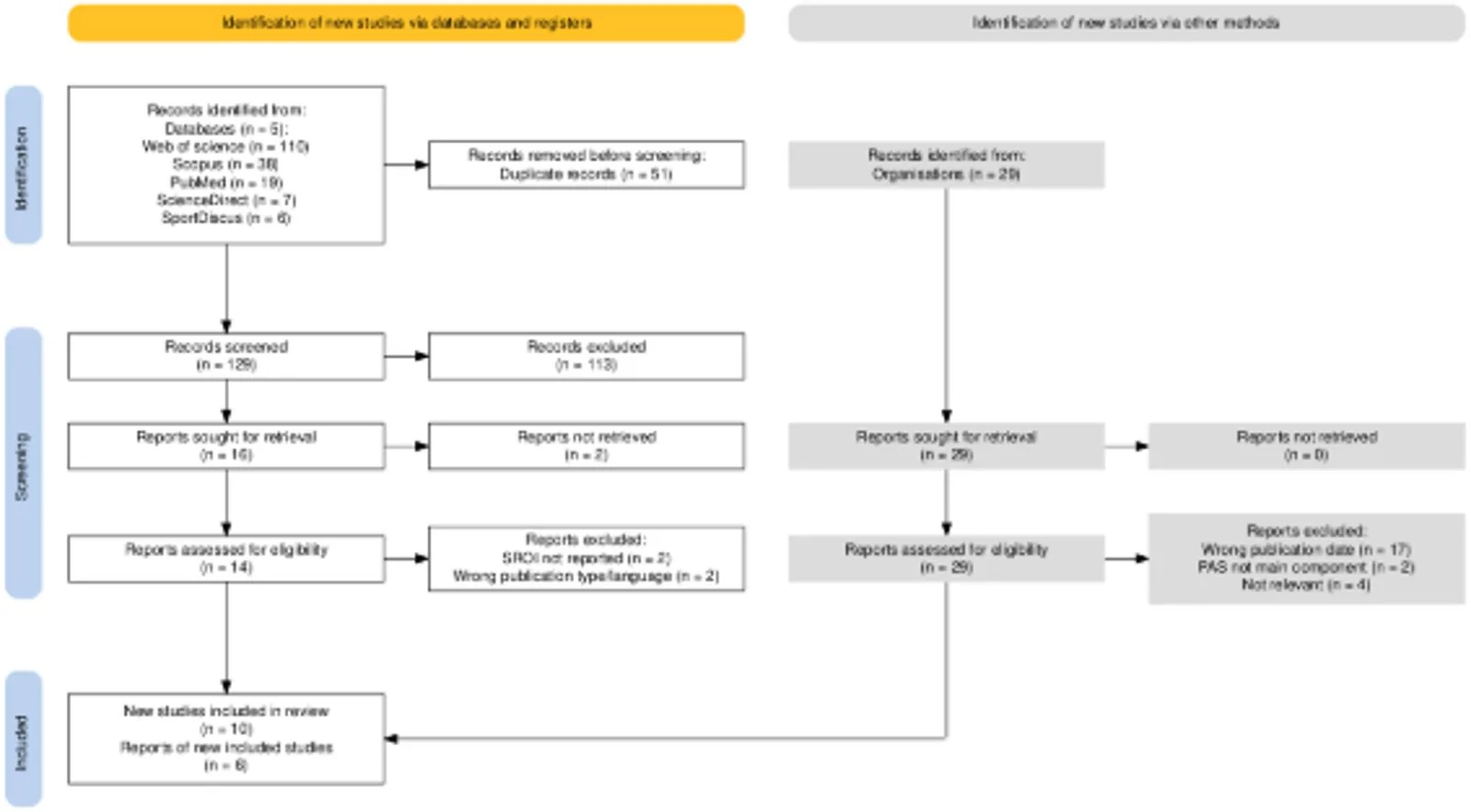
PRISMA flow diagram for study selection

A systematic search of five databases – Web of Science (*n* = 110 records), Scopus (*n* = 38), PubMed (*n* = 19), ScienceDirect (*n* = 7), and SportDiscus (*n* = 6) – initially identified 180 records. After removing 51 duplicate records using automation tools, 129 unique records remained for title and abstract screening. We screened these and excluded 113 records that clearly did not meet the inclusion criteria. Reasons for exclusion at each stage of the screening process are presented in the PRISMA flow diagram (Fig. 1). We sought to retrieve 16 records for full-text review; 2 could not be obtained, leaving 14 reports to assess for eligibility. Upon full-text assessment, 4 reports were further excluded (2 did not report an SROI ratio, and 2 were excluded for being the wrong publication type or language), yielding a final set of 10 SROI studies that met all inclusion criteria.

In addition, 29 records were identified through other sources (via Social Value UK), and all were retrieved for full-text screening; however, only 6 of these met the inclusion criteria (17 were excluded for being outside the eligible publication date range, 2 for not having sport or physical activity as a main component, and 4 for not being relevant to the review).

Finally, 16 studies were included in the review – from these studies, 62.5% (*n* = 10) are from academic search, while 37,5% (*n* = 6) from grey literature. The study selection process is summarized in the PRISMA flow diagram (Fig. 1).

### Data extraction and synthesis

A structured template for data extraction was created using Microsoft Excel to maintain uniformity across the studies included. Two reviewers independently extracted data from each qualifying study, resolving any discrepancies through discussion and, if needed, by consulting a third reviewer. Extracted data included study characteristics (author, year, country, publication type), intervention characteristics (type, target population, sample size, duration), economic evaluation details (costs, outcomes, SROI ratio), and methodological quality indicators required for the Krlev et al. framework [28].

The extracted data were organized into a table (see Table 2) to offer a comparative overview of the included studies. After extraction, data synthesis was conducted in three stages: Initially, in descriptive synthesis, study characteristics and findings were summarized narratively to identify patterns in intervention type, population focus, outcome domains, and reported SROI values. Studies were categorized by intervention type (e.g., preventive vs. development programs) and by publication type (peer-reviewed vs. grey literature) to examine differences in scope and reported returns. Preventive programs aim to reduce the risk or progression of health-related conditions (e.g. chronic diseases), whereas sport-for-development programs primarily target broader social outcomes such as inclusion, empowerment, and community development.

Subsequently, for quantitative synthesis, the authors aimed to provide an aggregate estimate of social return through a meta-analysis, as described below. Finally, a conceptual framework was developed to synthesize outcomes across health, social, economic, and sport-specific domains at both individual and community levels. This framework was iteratively derived from the extracted data and used to illustrate the multidimensional nature of value generated by physical activity and sport interventions.

### SROI reporting quality checklist – a quality assessment

To evaluate the methodological quality of the studies, we used a 12-item SROI reporting quality checklist developed by Krlev et al. [28]. The checklist assesses key aspects of SROI methodology, including stakeholder identification, transparency of financial proxies, justification of deadweight and attribution assumptions, sensitivity analysis, documentation of data sources, and critical reflection on study limitations. Each criterion is scored dichotomously (met = 1, not met = 0), resulting in a total score ranging from 0 to 12. This checklist was used earlier also in several SROI studies [17, 19, 29, 30]. Two reviewers independently evaluated the methodological quality of the included studies using the Krlev et al. framework. Accordingly, the SROI reporting quality checklist should be interpreted as an assessment of reporting quality and methodological transparency, rather than as a full evaluation of the validity of the underlying valuation assumptions or as a formal risk-of-bias assessment. In cases of disagreement, discrepancies were resolved through discussion, and the third reviewer made the final decision when consensus could not be reached. Scores were used descriptively as indicators of SROI reporting quality and methodological transparency rather than as a formal risk-of-bias assessment, and no study was excluded on the basis of its quality score. The full list of the 12 quality assessment criteria, along with the scores assigned to each study, is provided in Additional File 3. Scores were determined based on a detailed qualitative assessment of each study.

We extracted SROI quality assessment scores from three sets of studies: (a) the grey-literature articles included in the earlier systematic review, (b) the grey-literature sources newly identified in our review, and (c) the peer-reviewed studies newly identified in our review. To assess whether mean quality scores differed across these three groups, we conducted independent two-sample t-tests assuming equal variances for every possible pair of groups, using a two-tailed significance level of 0.05.

### Quasi meta-analysis

Due to significant data limitations in the retrieved studies, a formal variance-weighted meta-analysis was not feasible. The quantitative synthesis was therefore conducted as an exploratory quasi meta-analytic summary of reported SROI ratios. Specifically, nearly all studies failed to report standard deviations (SDs) or confidence intervals (CIs) associated with their effect estimates. This is partly due to the nature of SROI methodology, where estimates are typically derived from modelling approaches and monetisation of outcomes rather than statistical inference. However, the absence of such measures limits the ability to assess uncertainty and to appropriately weight study findings in a meta-analytical framework. Attempts to contact the authors for this critical information did not yield responses. Consequently, we proceeded with an alternative analytical approach involving the calculation of both unweighted and sample size-weighted mean effect sizes.

The unweighted descriptive mean was computed as a simple arithmetic average across individual study means, as follows:

$$\:{\stackrel{-}{x}}_{\mathrm{meta}}=\:\frac{{\sum\:}_{i=1}^{k}{\overline{x}}_{i}}{\mathrm{k}}$$

The sample-size-weighted descriptive mean was calculated using each study’s sample size to provide greater influence on studies with larger participant numbers:

$$\:{\stackrel{-}{x}}_{\mathrm{meta-weighted}}=\:\frac{{\sum\:}_{i=1}^{k}({\overline{x}}_{i}\times\:{n}_{i})}{{\sum\:}_{i=1}^{k}{n}_{i}}$$

Given the lack of variance-related data, these methods were used as transparent descriptive summaries of the available evidence, but they do not account for between-study heterogeneity or precision. Given the methodological heterogeneity and lack of variance data, this meta-analytic synthesis is strictly exploratory, serving to map available trends rather than establish definitive statistical comparisons.

To describe heterogeneity in reported SROI ratios, we calculated the number of estimates, mean, median, and range within subgroups defined by publication type and intervention type. These analyses were conducted at the level of reported SROI estimates. No formal subgroup tests were performed because the number of estimates was small and within-subgroup methodological heterogeneity was substantial.

In addition, correlation analyses were performed to examine relationships among key study characteristics, specifically the intervention duration (time horizon in weeks), sample size, total intervention costs (in euros), and the social return on investment (SROI) ratio. Pearson’s correlation coefficients were calculated to quantify the strength and direction of linear relationships among these variables. Correlations were based on pairwise complete observations and are reported with p-values, 95% confidence intervals, and pairwise sample sizes. Because one study reported two distinct SROI estimates, the quantitative synthesis was conducted at the level of reported SROI estimates rather than studies.

## Results

### Study characteristics

In the systematic review, sixteen studies that met the inclusion criteria were examined, covering publications from 2018 to 2024. There was a significant rise in the number of studies in recent years, with more than two-thirds being published from 2020 onwards. Of these, ten were peer-reviewed academic studies [21, 31–39], while six were reports from the grey literature [40–45], such as consulting or organizational evaluations. The majority of the research was concentrated in Europe, with Wales contributing four studies, and others originating from England, Ireland, Italy, the Netherlands, and Turkey. Outside of Europe, only a few studies were found, with one each from Japan [38] and Taiwan [43]. No studies were identified from North America, Africa, or Australia, highlighting a geographic gap in research.

The interventions assessed varied widely in physical activity contexts but were generally classified as either (primary or secondary) preventive interventions [31, 33–37, 39, 40] or sport-for-development programs [32, 38, 42, 43]. The remaining studies focused on high-performance sport [21], sport for empowerment [41], “tertiary” prevention [44], and general “sport for all” community activities [45], with one study each. The target populations were diverse, including general community members [31, 34, 36, 40, 45], schoolchildren and youth, including children with disabilities or disadvantaged adolescents [32, 37, 41–43], older adults, clinical populations, such as patients with early dementia [33, 35, 39, 44], and even stakeholders of sports clubs [21, 39].

Across reported SROI estimates, sample sizes ranged from 19 to 16,106 participants, indicating both small pilot interventions and large-scale community initiatives. Most analyses were evaluative [31, 33–42, 44, 45], meaning that SROI calculations were based on observed outcomes measured during or after programme implementation, rather than predictive studies [21, 32, 43], which estimate future social value based on projected or modelled outcomes. The three predictive estimates were 2.98:1, 3.26:1, and 12.49:1. These values do not suggest a consistent tendency for predictive SROI studies to report higher ratios than evaluative studies, although the small number of predictive estimates precludes any formal inference. Table 2 provides a summary of the key characteristics and results of each included study.

**Table 2:** Summary of included studies (*N* = 16) evaluating social return on investment (SROI) of physical activity interventions

Ref	Study (Author, Year)		Country	Intervention Type	Sample Size	SROI Ratio
[31]	Davies et al. (2021)	Academic	England	Preventive – primary	16,106	1.20:1
					1224	3.42:1
[32]	Di Francesco et al. (2023)	Academic	Italy	Sport for development	19	3.26:1
[33]	Hartfiel et al. (2022)	Academic	United Kingdom	Preventive – secondary	60	5.94:1
[34]	Hartfiel et al. (2023)	Academic	Wales	Preventive – secondary	74	2.87–4.67:1
[35]	Jones et al. (2020)	Academic	Wales	Preventive – secondary	104	5.07:1
[21]	Lombardo et al. (2019)	Academic	Italy	High-performance sport	*Not reported*	2.98:1
[36]	Makajnuola et al. (2023)	Academic	Wales	Preventive – primary	52	4.37–5.36:1
[37]	Oosterhoff et al. (2020)	Academic	Netherlands	Preventive – primary	1676	0.03:1
[38]	Oshimi et al. (2021)	Academic	Japan	Sport for development	3450	5.3:1
[39]	Skinner et al. (2023)	Academic	Wales	Preventive – secondary	24	4.67:1
[40]	Whitebarn Consulting (2019)	Grey literature	Ireland	Preventive – primary	511	15.3:1
[41]	Özgün (2021a)	Grey literature	Turkey	Sport for empowerment	83	8.8:1
[42]	Özgün (2023)	Grey literature	Turkey	Sport for development	201	7.24:1
[43]	KPMG Sustainability Consulting (2021)	Grey literature	Taiwan	Sport for development	574	12.49:1
[44]	Whitebarn Consulting (2020)	Grey literature	Ireland	Tertiary prevention	255	22.32:1
[45]	Özgün (2021b)	Grey literature	Turkey	Sport for all activities	1130	101.05:1

Primary prevention refers to preventing the onset of conditions; secondary prevention to early-stage intervention; tertiary prevention to managing existing conditions; sport-for-development refers to interventions targeting social outcomes such as inclusion and empowerment

These studies examined a broad spectrum of outcomes, highlighting the diverse effects of physical activity programs. Health-related outcomes were a major focus [21, 31–37, 39, 40, 43–45], with many interventions monitoring shifts in physical health (such as self-reported health and health-related quality of life) or health behaviours (like the frequency of physical activity). Almost all studies evaluated mental health and well-being [32, 34, 36, 39, 45], noting enhancements in mental well-being, confidence [33, 35, 41, 43, 45], and self-esteem [21].

Several studies also documented social outcomes at both the individual and community levels. Individual social outcomes included increased social connections, improved social skills or trust, and greater inclusion of marginalized groups [32–36, 43, 44]. On a societal scale, some studies assessed outcomes like education (e.g., school absenteeism) [21, 31, 37, 40, 43] and crime rates [31], along with community cohesion indicators such as social trust [34, 36] and volunteering [40, 45]. In [40, 45] volunteering was also identified as an important mechanism through which sport and physical activity interventions may strengthen wellbeing, social connectedness, and community social capital.

Notably, some evaluations considered outcomes related to community development and economic benefits. For example, one high-performance sport program assessed tourism and city appeal, local revenue growth, and the enhancement of the city’s image [21], while others observed reduced health service use [34, 36] or increased participation in local facilities [35]. This wide range of outcomes demonstrates that physical activity interventions were considered from a holistic perspective, capturing benefits that extend from personal health improvements to broader social value, including civic engagement and economic contributions to the community.

Regarding social return on investment outcomes, nearly all estimates showed a net positive return. Across the 17 extracted SROI estimates from 16 studies, 16 exceeded 1:1, indicating that monetized social benefits surpassed programme costs. SROI ratios varied widely, from 0.03:1 to 101.05:1. For instance, an SROI of 3.42:1, as reported by a community sport program in England, implies that for every £1 invested, about £3.42 of social value was generated. Most SROI values were in the single digits, with over half of the studies yielding ratios between approximately 3:1 and 6:1.

The median SROI across all studies was around 5:1, indicating that a fivefold return on investment was a common outcome for physical activity initiatives. However, there were significant outliers at both extremes. On the lower end, a school-based physical activity program in the Netherlands yielded a nearly neutral return (0.03:1), essentially showing no significant social gain for the investment in that context. Conversely, exceptionally high returns were noted in a few cases: an Irish “tertiary prevention” program focused on health and social well-being achieved about 22:1, and a municipal sport-for-all initiative in Turkey reported an SROI of 101:1—an extraordinarily large benefit relative to cost.

### Quality assessment

**Fig. 2:**
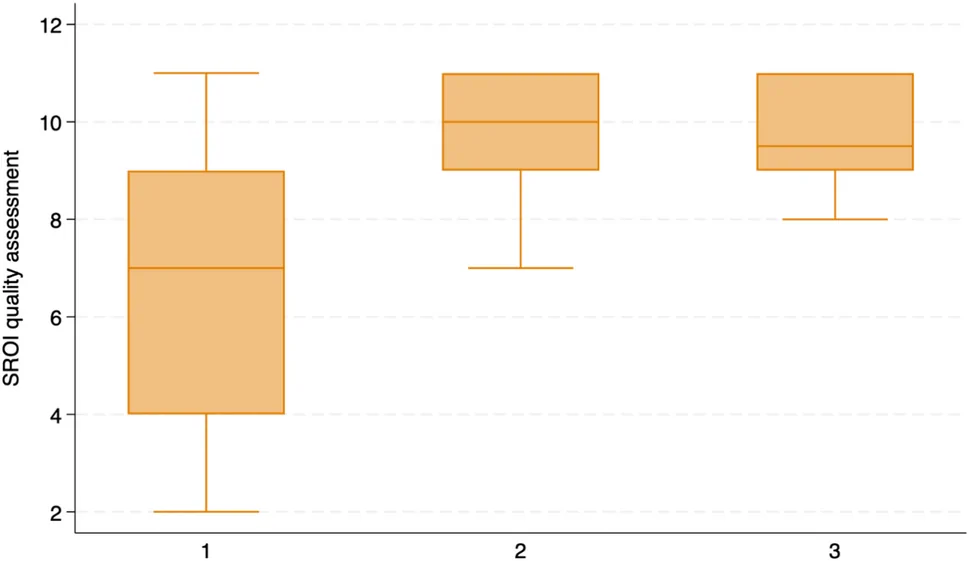
Box plots of quality scores across groups. Note: Scores refer to the SROI reporting quality checklist. Numerical labels indicate group membership: (1) grey-literature articles included in the earlier systematic review; (2) grey-literature sources newly identified in our review; and (3) peer-reviewed studies newly identified in our review

Figure 2 presents box plots of SROI quality scores across three groups. The numerical markers denote: [1] grey-literature articles from the earlier systematic review [2], newly identified grey-literature sources in our review, and [3] newly identified peer-reviewed studies.

The mean SROI quality score for the earlier grey literature was 6.53 (SD = 2.74), whereas our newly identified grey-literature and peer-reviewed groups scored an average of 9.67 (SD = 1.63) and 9.60 (SD = 1.17), respectively. Pairwise comparisons revealed that both of our new groups achieved significantly higher quality scores than the earlier grey literature (*p* = 0.016 for earlier vs. new grey; *p* = 0.003 for earlier vs. peer-reviewed), while there was no significant difference between our grey-literature and peer-reviewed samples (*p* = 0.926).

### Meta-analysis

Due to methodological limitations in the source studies, the quantitative synthesis employed both unweighted and sample-size-weighted mean calculations. Across the 17 SROI estimates, the unweighted mean was 12.22:1 and the median was 5.07:1. The sample-size-weighted mean, calculated for the 16 estimates with available sample size, was 7.07:1. Sensitivity analyses showed that these estimates were strongly influenced by the highest SROI value of 101.05:1. Excluding this value reduced the unweighted mean to 6.67:1 and the weighted mean to 2.72:1. Excluding the two highest SROI values further reduced the unweighted and weighted means to 5.62:1 and 2.51:1, respectively. These findings indicate that mean-based summaries, particularly the weighted mean, are sensitive to extreme values; therefore, the median and sensitivity-adjusted estimates provide a more conservative summary of the evidence base. Full sensitivity results are provided in Additional File 4.

To examine heterogeneity descriptively, we summarised reported SROI estimates by publication type and intervention type (Table 3). These subgroup summaries were calculated at the level of reported SROI estimates and are intended to describe patterns in the available evidence rather than to support formal subgroup inference.

**Table 3:** Descriptive subgroup summaries of reported SROI estimates by publication type and intervention type

Panel	Subgroup	*N*	Mean	Median	Range	Min	Max
Publication type	Peer-reviewed	11	3,68	3,77	0.03–5.94	0,03	5,94
Publication type	Grey literature	6	27,87	13,90	7.24-101.05	7,24	101,05
Intervention type	Preventive/targeted health	10	6,66	4,77	0.03–22.32	0,03	22,32
Intervention type	Sport for development/empowerment	5	7,42	7,24	3.26–12.49	3,26	12,49
Intervention type	Sport for all	1	101,05	101,05	101.05-101.05	101,05	101,05
Intervention type	High-performance sport	1	2,98	2,98	2.98–2.98	2,98	2,98

Table 3 shows that reported SROI estimates were generally lower and less variable in peer-reviewed publications than in the grey literature. Across intervention types, preventive/targeted health and sport for development/empowerment had broadly comparable central estimates, while the markedly higher value for sport for all was based on a single estimate. Given the small number of estimates in several subgroups and the heterogeneity of valuation assumptions, these patterns should be interpreted as descriptive rather than as rankings of intervention effectiveness.

Pearson correlations were based on pairwise complete observations, with sample sizes ranging from 14 to 17 across variable pairs (Table 4). The analysis did not reveal a stable pattern of association between SROI ratios and study-level characteristics. For all SROI-related pairs, confidence intervals were wide and crossed zero, indicating that the available data do not support conclusions about population-level relationships between SROI ratios and intervention duration, sample size, or intervention cost. Accordingly, these correlations are reported only as exploratory descriptive diagnostics. Overall, while interventions appear economically beneficial, results highlight substantial variability and only limited predictive value of duration, sample size, and cost in explaining differences in reported social returns. Interpretation of these findings should account for methodological constraints and variability inherent in the reviewed studies.

**Table 4:** Pearson correlations between study characteristics and SROI estimates

Variable pair	*n*	Pearson *r*	95% CI	*p*-value
Time horizon – Sample size	14	-0.21	[-0.66, 0.36]	0.478
Time horizon – Cost	15	-0.18	[-0.63, 0.37]	0.519
Time horizon – SROI ratio	15	0.02	[-0.49, 0.53]	0.931
Sample size – Cost	16	0.96	[0.88, 0.99]	< 0.001
Sample size – SROI ratio	16	-0.10	[-0.57, 0.42]	0.708
Cost – SROI ratio	17	-0.16	[-0.60, 0.35]	0.534

Correlations are based on pairwise complete observations. The unit of analysis is the reported SROI estimate; one study contributed two distinct SROI estimates. Pairwise n varies because of missing information on time horizon, sample size, or cost

## Discussion

Overall, the reviewed studies suggest that investments in physical activity and sport generally generate positive social value across diverse intervention contexts. Despite substantial methodological and contextual heterogeneity, most studies reported benefits exceeding programme costs, supporting the role of physical activity interventions as potentially valuable public and community investments. Compared with the earlier review by Gosselin et al. [19], which relied predominantly on grey literature, our review incorporates a growing body of peer-reviewed evidence published after 2018. The newly identified evidence, including both peer-reviewed and grey-literature studies, generally showed higher reporting quality than the earlier grey-literature evidence reviewed by Gosselin et al. This strengthens confidence that recent SROI studies in this field are becoming more transparent, although comparability remains limited by differences in valuation assumptions and outcome scope. However, the findings also reveal a wide range in SROI magnitudes, highlighting important nuances. The variation (from almost no return in one instance to extremely high returns in others) suggests that contextual factors and program design are likely crucial in determining the extent of social value realized. For example, the single study [37] that reported a minimal return (0.03:1) involved a school-based intervention with a limited two-year timeframe—a context where benefits might take longer to manifest, where the monetised outcome scope was narrower than in broader community-based SROI studies, or where short-term measurement did not fully capture delayed health, educational, or wellbeing effects. Conversely, programs targeting broad outcomes or vulnerable populations tended to report higher returns.

The exceptionally high SROI ratio (101:1) reported in the Turkish municipal sport-for-all programme likely reflects a combination of methodological and contextual factors [45]. It monetised a broad range of subjective wellbeing and social capital outcomes across multiple stakeholder groups, many of which were intangible and closely interrelated. Although such outcomes are central to SROI methodology, extensive reliance on self-reported wellbeing valuations may increase the risk of overlapping or inflated value estimates, particularly when attribution and counterfactual assumptions are difficult to verify. In addition, the allocation of organizational investment cost across stakeholder groups may have contributed to a relatively low denominator compared with the breadth of benefits included in the analysis. The 101:1 estimate was retained because it met the inclusion criteria. However, we consider it more plausibly an influential valuation-scope outlier than a generalisable benchmark for sport-for-all programmes. This interpretation is supported by the breadth of monetised wellbeing and social-capital outcomes, the reliance on intangible and potentially overlapping benefits across stakeholder groups, and the relatively low cost denominator compared with the scope of benefits included. Under more conservative or harmonised valuation assumptions, this estimate would likely be materially lower. For this reason, the median and sensitivity-adjusted estimates should be treated as the more cautious summaries of central tendency in the current evidence base.

Similarly, the high SROIs reported in the Irish cases (15:1 and 22:1) suggest that preventive health programs addressing multiple dimensions of well-being (physical, social, and economic) can deliver significant value, especially when implemented on a large scale [40, 44]. To further interpret these outliers, differences in methodological choices should be considered. Studies including a broader range of monetised outcomes (e.g., health, wellbeing, and social effects) and applying less conservative assumptions regarding attribution or deadweight tend to report higher SROI values, while narrower outcome scopes and shorter time horizons are associated with lower ratios. Therefore, variation in SROI estimates largely reflects differences in analytical scope and assumptions, rather than directly comparable effect sizes across studies. When comparing intervention types, preventive and targeted health programmes tended to report more consistent and moderate SROI values, whereas broader community-based or sport-for-development initiatives were associated with higher, but more variable, returns. These findings should be interpreted as descriptive patterns within the available SROI evidence base rather than as definitive rankings of intervention effectiveness, because intervention categories differed substantially in methodological scope, valuation assumptions, and reporting practices.

Across studies, improvements in physical health were frequently accompanied by gains in wellbeing, confidence, social connectedness, and broader community outcomes. These findings highlight the multidimensional nature of value generated through physical activity interventions and demonstrate how SROI can capture benefits extending beyond direct health effects. Despite the generally positive findings, we also identified several gaps and inconsistencies in the evidence base that need attention. Geographically, research on the social return of physical activity and sport is uneven. Most of the included studies were conducted in Europe, with very limited representation from other regions. The concentration of studies in Wales may partly reflect the country’s distinctive policy environment, particularly the Well-being of Future Generations (Wales) Act 2015, which places strong emphasis on preventive public health approaches and broader social value creation [46]. Consequently, the relatively high number of Welsh SROI evaluations may not only indicate greater research activity, but also a policy context that is especially supportive of social impact assessment frameworks, which should be considered when interpreting the generalizability of the findings. This indicates a need for more studies in under-represented areas (such as North America, Asia beyond Japan/Taiwan, Africa, and Latin America) to determine if the strong SROI observed in European contexts is applicable elsewhere. Different populations and healthcare systems may produce different outcomes, and cultural factors could affect the social benefits realized through sport and exercise. Expanding the geographic scope of SROI studies will enhance the generalizability of conclusions and help identify context-specific factors that influence social return.

A significant gap exists in the methodological rigor and standardization of SROI evaluations. The studies reviewed showed considerable variation in how they addressed factors such as deadweight, attribution, and whether they included any uncertainty or sensitivity analysis. Notably, most studies did not provide confidence intervals or other statistical uncertainty measures for their SROI values (except [21, 37, 43], making it challenging to assess the precision and reliability of these estimates. Some reports explicitly mentioned deadweight adjustments, often using estimates or ranges [21, 32, 34–36, 39–45], while others either ignored it or assumed zero deadweight, attributing all observed outcomes to the intervention [31, 33, 37, 38]. This inconsistency implies that although the overall effect (benefits outweighing costs) is generally positive, the precise magnitude of the SROI should be interpreted cautiously and within the context of each study’s assumptions.

One of our key research questions concerned whether peer-reviewed and grey literature studies differ systematically in their reported SROI. While our analysis shows that peer-reviewed studies generally report more conservative SROI ratios, their reporting quality and methodological transparency, as measured by the SROI reporting quality checklist, were comparable to those of the newly identified grey-literature sources and higher than those observed in the earlier. This contrast may help explain why earlier reviews, which drew heavily on grey literature, reported somewhat higher average SROI values than we observed here. Rather than critiquing individual studies, this finding underscores the need for field-wide reporting standards and harmonised SROI guidelines in physical activity research. These standards could include consensus on outcome measures, valuation techniques (e.g., monetizing improvements in well-being or social capital), and sensitivity analysis reporting. Enhancing methodological consistency will boost the credibility of SROI findings and facilitate easier comparison across studies.

Table 5 summarizes the main health, social, economic, and sport-specific outcomes identified across the reviewed studies at both individual and community levels. It also reveals a consistent trend: interventions yield similar advantages for both individuals and their communities. On a health level, personal improvements lead to broader system-level outcomes, like less pressure on healthcare services and the encouragement of healthier lifestyles. The social aspect highlights how personal advancements in confidence, social skills, and self-awareness are reflected in increased community trust, cohesion, and a sense of belonging. The economic aspect shows a dual benefit: individuals experience higher educational achievements, job satisfaction, and career opportunities, while organizations and societies benefit from increased revenues, reduced productivity losses, and public savings. These results bolster the case for viewing sport and physical activity as not only health-promoting but also economically strategic investments. Lastly, the sport-specific aspect, though more focused, shows the direct acquisition of knowledge and skills, which can lay the groundwork for sustained engagement in active lifestyles.

**Table 5:** Key outcome groups of physical activity and sport interventions

Dimension	Individual	Community/social
Health	Better overall health	Less strain on healthcare system
	Reduced risk of NCDs	
	Improved subjective wellbeing and mental well-being	Promotion of physical activity and healthy lifestyle
	Improved physical well-being	
Social	Improved self-confidence	Improved social capital (e.g. social trust, cohesion, volunteering)
	Improved social connectedness and interpersonal relationships	
		Increased feeling of belonging
	Improved social and interpersonal skills	Improved social life
		Improved family and friendship relationships
	Increased self-awareness and motivation	Increased image and prestige of the community/organization
		Reduction in criminal incidents
Economic	Higher educational attainment	Increase in revenue and profit of the organization
	Higher starting salary	Increase in brand and attractiveness of community/organization
	Higher job satisfaction	Reduced productivity losses and workforce burden
	Contribution to career	Public money savings
Sport specific	Improved sport specific knowledge and skills	-

### Limitations

Several limitations should be acknowledged. First, the evidence base remains small, comprising 16 studies and 17 reported SROI estimates. As a result, the subgroup summaries and correlation analyses are underpowered and should be interpreted as descriptive rather than inferential. Second, the studies are geographically concentrated in Europe, with a notable cluster from Wales, which may limit the generalisability of findings to other policy, health-system, and sport-sector contexts. Third, we relied on SROI ratios reported by the original studies and could not independently verify or re-estimate the underlying valuation models. Fourth, substantial heterogeneity in outcome scope, financial proxies, time horizons, deadweight, attribution, displacement, and sensitivity assumptions prevented harmonised adjustment across studies. Finally, the Krlev et al. instrument was used as an SROI reporting quality checklist, not as a full risk-of-bias assessment or validation of the underlying monetary valuations.

## Conclusions

This study provides the first systematic review with an exploratory quasi meta-analytic synthesis of SROI in sport and physical activity that combines both peer-reviewed and grey literature since Gosselin et al. [19]. By adding peer-reviewed studies and applying a meta-analytic approach, we extended the evidence base and descriptively compared whether peer-reviewed and grey-literature sources differ in their reported returns.

Our findings confirm that investments in physical activity generally generate positive social value, with median and sensitivity-adjusted estimates suggesting returns of several times the initial costs, although mean-based estimates are sensitive to extreme values. Peer-reviewed studies reported more cautious SROI ratios, while recent peer-reviewed and grey-literature studies both showed improved reporting quality compared with earlier grey-literature evidence. At the same time, variation across programs and methods underscores the importance of context and careful interpretation of SROI figures.

Overall, the findings support physical activity interventions as socially valuable investments, although interpretation of SROI magnitudes requires careful consideration of methodological assumptions and contextual differences. To move the field forward, greater methodological consistency and broader geographic coverage are needed, alongside regular evidence updates, so that policymakers can rely on the most robust and current knowledge when making investment decisions. Future research should build on these insights by addressing the noted gaps to ensure that the social impact of physical activity is fully understood and optimally harnessed. 

## Supplementary Information

Supplementary Material 1.

Supplementary Material 2.

Supplementary Material 3.

Supplementary Material 4.

Supplementary Material 5.

## Data Availability

All data supporting the findings of this study are provided in the article and its supplementary files.

## Abbreviations

CBA: Cost–Benefit Analysis
CEA: Cost–Effectiveness Analysis
CI: Confidence Interval
NCD: Non–Communicable Disease
PAS: Physical activity and sport
PICO: Population, Intervention, Comparison, Outcome Framework
PRISMA: Preferred Reporting Items for Systematic Reviews and Meta–Analyses
Ref: Reference (of selected study)
SD: Standard Deviation
SROI: social return on investment framework
WHO: World Health Organization
