# Supplementary material for: Do investments in sport pay off: a systematic review and exploratory quasi meta-analysis of social return on investment in sport and physical activity
Source: Int J Behav Nutr Phys Act. 2026 Jul 27;23:99. doi: 10.1186/s12966-026-01957-2 (PMC13693046; doi:10.1186/s12966-026-01957-2)
Supplement: Supplementary file 1 [file 12966_2026_1957_MOESM4_ESM.pdf]

Table A. Sensitivity analysis excluding extreme SROI values

Analysis	SROI estimates, n	Estimates with sample size, n	Total sample weight	Unweighted mean	Weighted mean	Median	Range
All estimates	17	16	25,543	12.22	7.07	5.07	0.03–101.05
Excluding highest SROI value	16	15	24,413	6.67	2.72	4.97	0.03–22.32
Excluding two highest SROI values	15	14	24,158	5.62	2.51	4.87	0.03–15.30
