# Supplementary material for: Do investments in sport pay off: a systematic review and exploratory quasi meta-analysis of social return on investment in sport and physical activity
Source: Int J Behav Nutr Phys Act. 2026 Jul 27;23:99. doi: 10.1186/s12966-026-01957-2 (PMC13693046; doi:10.1186/s12966-026-01957-2)
Supplement: Supplementary file 2 [file 12966_2026_1957_MOESM5_ESM.pdf]

Summary of the SROI Calculation Process

Social Return on Investment (SROI) is a framework used to evaluate the broader social, economic, and health-related value generated by an intervention relative to the resources invested. Unlike traditional cost-benefit or cost-effectiveness analyses, SROI explicitly incorporates non-market outcomes such as wellbeing, social cohesion, confidence, social inclusion, and community engagement into economic evaluation. In sport and physical activity research, SROI is particularly valuable because interventions often generate multidimensional benefits that extend beyond direct healthcare savings (Banke-Thomas et al., 2015, Gosselin et al., 2020)

The SROI Process and Its Sequential Stages

The attached SROI framework illustrates the six major stages of the SROI process (Banke-Thomas et al., 2015). The first three stages mainly focus on data collection and stakeholder engagement, while the middle stages combine data collection with analytical modelling, and the final stage concerns dissemination and integration of findings into decision-making.

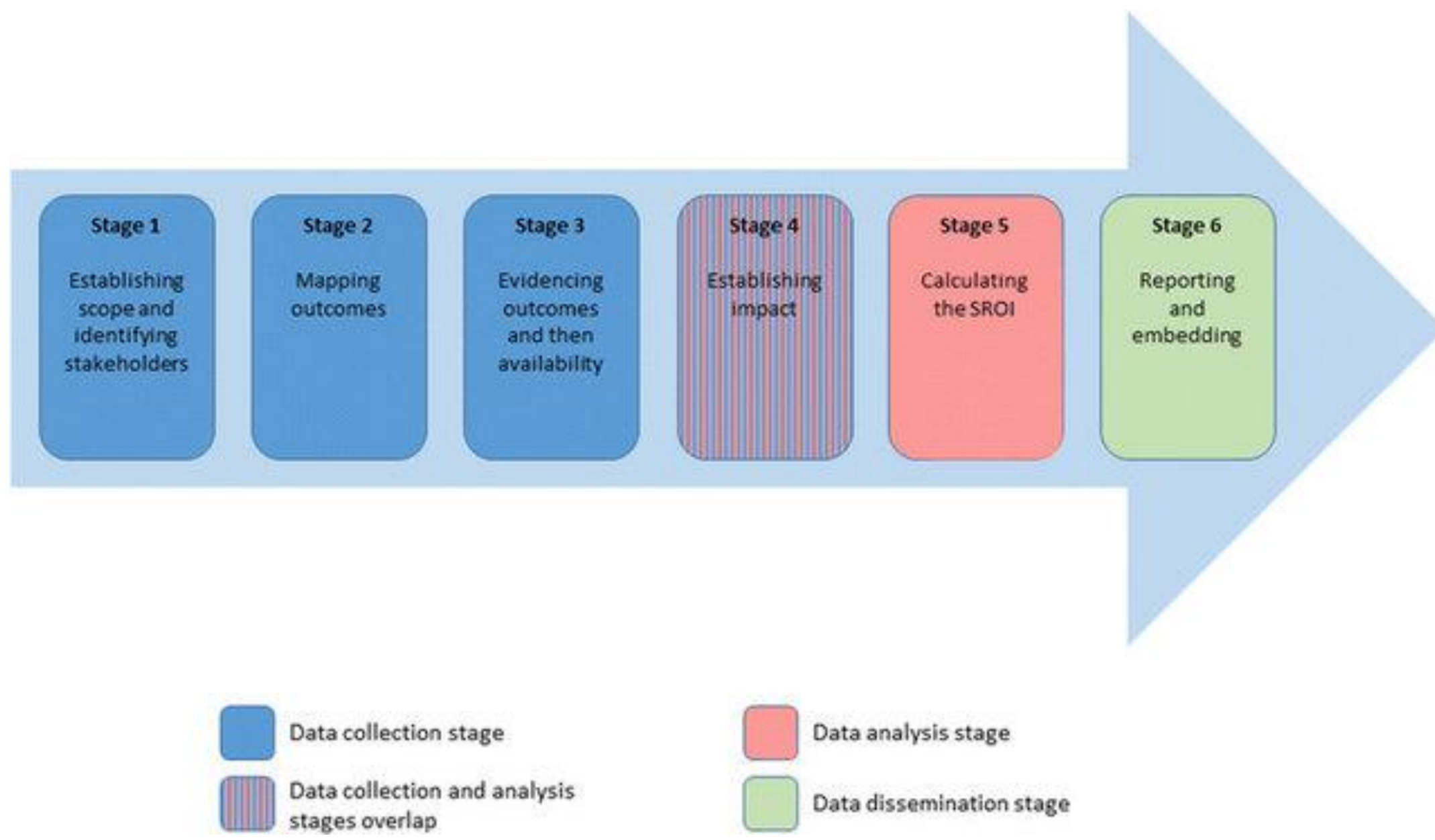

Figure 1. Main stages of the Social Return on Investment (SROI) process (Banke-Thomas et al., 2015)

Stage 1 – Establishing scope and identifying stakeholders

The evaluation begins by defining the scope, objectives, timeframe, and stakeholders involved in the intervention. Stakeholders may include participants, families, healthcare providers, local communities, employers, or public institutions.

Stage 2 – Mapping outcomes

This phase identifies intended and unintended outcomes experienced by stakeholders. Outcome mapping is commonly conducted using interviews, surveys, focus groups, programme reports, and logic models (King, 2014, Davies et al., 2019).

Stage 3 – Evidencing outcomes and assigning values

At this stage, outcomes are supported with empirical evidence and monetised using financial proxies. Common proxies include Quality Adjusted Life Years (QALYs), avoided healthcare costs, reduced absenteeism, wellbeing valuation databases, and social value banks. This step is critical because SROI relies heavily on the validity and transparency of financial proxy selection (Banke-Thomas et al., 2015, Ashton et al., 2024).

Stage 4 – Establishing impact

The impact estimation phase adjusts gross outcomes to avoid overestimating social value. Several key methodological parameters are applied here, including deadweight, attribution, displacement, and drop-off. Deadweight estimates the proportion of outcomes that would have occurred anyway without the intervention. Attribution measures how much of the observed impact can genuinely be linked to the programme rather than external actors. Displacement assesses whether positive outcomes merely replace benefits elsewhere, while drop-off estimates how benefits diminish over time. This stage directly addresses one of the major concerns raised in SROI literature regarding comparability and credibility of SROI ratios (Gosselin et al., 2020, Ashton et al., 2024).

Stage 5 – Calculating the SROI

After adjusting outcomes and discounting future benefits where necessary, the total adjusted social value is divided by the total investment cost. The resulting ratio expresses how much social value is generated for every monetary unit invested.

Stage 6 – Reporting and embedding

The final phase involves transparent reporting, sensitivity analyses, stakeholder feedback, and integration of findings into organisational decision-making. Recent literature increasingly emphasises the importance of methodological transparency, especially regarding valuation assumptions and uncertainty analyses (Ashton et al., 2024).

Standard SROI Formula

SROI Ratio = Present Value of Total Social Benefits / Total Investment Cost

An SROI ratio of 5:1 means that every unit of investment generates approximately five units of social value. However, SROI estimates may vary substantially depending on stakeholder scope, valuation methods, assumptions regarding deadweight and attribution, and the breadth of monetised outcomes.

Methodological Issues when calculating SROI

- Large variation in SROI estimates often results from differences in valuation assumptions and financial proxy selection (Gosselin et al, 2020).

- Studies applying broad wellbeing valuations and subjective social outcomes may report substantially higher SROI ratios.
- Failure to transparently report deadweight, attribution, displacement, or drop-off assumptions may reduce comparability between studies.
- Sensitivity analyses are essential because extreme SROI values can strongly influence pooled averages.
- Peer-reviewed studies often apply more conservative assumptions and therefore tend to report lower but methodologically stronger SROI ratios.

References

1. Banke-Thomas AO, Madaj B, Charles A, et al. Social Return on Investment (SROI) methodology to account for value for money of public health interventions: a systematic review. BMC Public Health. 2015;15:582.
2. Gosselin V, Boccanfuso D, Laberge S. Social return on investment (SROI) method to evaluate physical activity and sport interventions: a systematic review. International Journal of Behavioral Nutrition and Physical Activity. 2020;17(1):26.
3. Ashton K, Cotter-Roberts A, Clemens T, Green L, Dyakova M. Advancing the social return on investment framework to capture the social value of public health interventions. Public Health. 2024;226:122–127.
4. Davies LE, Taylor P, Ramchandani G, Christy E. Social return on investment (SROI) in sport: a model for measuring the value of participation in England. International Journal of Sport Policy and Politics. 2019;11(4):585–605.
5. King N. Making the case for sport and recreation services: The utility of social return on investment (SROI) analysis. International Journal of Public Sector Management. 2014;27(2):152–164.
